# Supplementary material for: Thermodynamic computing via autonomous quantum thermal machines
Source: Sci Adv. 2024 Sep 4;10(36):eadm8792. doi: 10.1126/sciadv.adm8792 (PMC11758477; doi:10.1126/sciadv.adm8792)
Supplement: Supplementary file 1 — Supplementary Text Fig. S1 [file sciadv.adm8792_sm.pdf]

Science Advances
AAAS

Supplementary Materials for

Thermodynamic computing via autonomous quantum thermal machines

Patryk Lipka-Bartosik *et al.*

Corresponding author: Patryk Lipka-Bartosik, patryk.lipka.bartosik@gmail.com

Sci. Adv. **10**, eadm8792 (2024)
DOI: 10.1126/sciadv.adm8792

This PDF file includes:

Supplementary Text
Fig. S1

A Details of the thermal NOT gate

The finite output reservoir $\mathcal{B}_z$ is initialised at some temperature $\beta_z(0)$ which changes according to Eq. (20). The total heat current that flows into this reservoir is a sum of two components, i.e. the currents given by Eqs. (17) and (19). More explicitly, the respective currents are given by

$$j_{\mathcal{C}} = \mu \epsilon_z \left[g_z(\beta_z(t)) - g_z(\beta_v) \right], \tag{S1}$$

$$j_{\mathcal{M}} = \mu' \epsilon_z \left[g_z(\beta_z(t)) - g_z(\beta_r) \right], \tag{S2}$$

After a sufficiently long time, the finite rezervoir $\mathcal{B}_z$ reaches the steady-state when $\dot{\beta}_z(t) = 0$, which happens precisely when

$$g_z(\beta_z^\infty) = \Delta g_z(\beta_v) + (1-\Delta) g_z(\beta_r), \tag{S3}$$

where $\Delta := \mu/(\mu + \mu')$ and β_z^∞ denotes the stationary value of $\beta_z(t)$. Eq. (S3) can be solved explicitly for β_z^∞, i.e.

$$\beta_z^\infty = \frac{1}{\epsilon_z} \log \left[\frac{1}{\Delta g_z(\beta_v) + (1-\Delta) g_z(\beta_r)} - 1 \right]. \tag{S4}$$

Let us now restrict it β_z^∞ to the range $[\beta_{\text{min}}, \beta_{\text{max}}]$ so that it can be interpreted as a logical signal. For that we enforce the additional constraints

$$\lim_{\beta_v \to \infty} g_z(\beta_z^\infty) = g_z(\beta_{\text{min}}), \qquad \lim_{\beta_v \to -\infty} g_z(\beta_z^\infty) = g_z(\beta_{\text{max}}), \tag{S5}$$

where we recall that $\beta_z^\infty = \beta_z^\infty(\beta_v, \beta_r, \Delta, \epsilon_z)$ and $\beta_v = \beta_v(\beta_0, \beta_1, \epsilon_z)$. The additional requirements from Eq. (S5) lead to the following set of equations.

$$\Delta + (1-\Delta) g_z(\beta_r) = g_z(\beta_{\text{min}}), \tag{S6}$$

$$(1-\Delta) g_z(\beta_r) = g_z(\beta_{\text{max}}), \tag{S7}$$

where $g_z(\beta_{\text{min}}) \geq g_z(\beta_{\text{max}})$. Solving these equations with substitution $\kappa := g_z(\beta_{\text{min}}) - g_z(\beta_{\text{max}}) \geq 0$ leads to $\Delta = (1-\kappa)/\kappa$ and $g_z(\beta_r) = g_z(\beta_{\text{max}})/(1-\kappa)$, or more precisely $\beta_r = \epsilon_z^{-1} \log[(1-\kappa)e^{\beta_{\text{max}}\epsilon} - \kappa]$. Plugging these values into Eq. (S4) and solving for β_z^∞ yields

$$\beta_z^\infty = \frac{1}{\epsilon_z} \log\left[\frac{1}{g_z(\beta_{\text{max}}) + g_z(\beta_v)\kappa} - 1\right]. \tag{S8}$$

The above equation describes the steady-state response of our inverter for input β_1. We plotted β_z^∞ as a function of the input temperature β_1 for the exemplary parameters in Fig. S1.

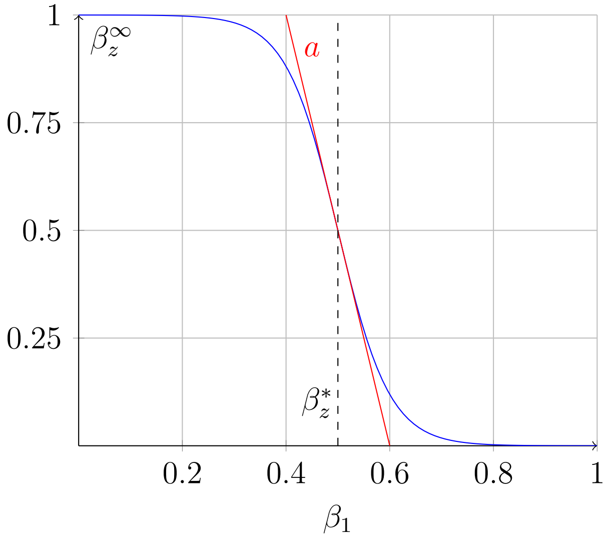

Figure S1: The response $\beta_z(\beta_1)$ of the machine as a function of the input temperature β_1. The plot was generated using the following values of parameters: $\epsilon_z = 0.5$, $\epsilon_0 = 20$, $\beta_0 = 0.5$, $\beta_{\text{min}} = 0$, $\beta_{\text{max}} = 1$.

The model has three free parameters that quantify its behaviour, namely ϵ_0, β_0 and ϵ_z. This parameters enter Eq. (S8) through the virtual temperature β_v. In order to characterize the behavior of our machine we now determine some properties of the response β_z^∞ as given by Eq. (S8). The threshold value β_z^* can be computed by finding the root of $\partial_{\beta_1}^2 \beta_z^\infty = 0$, i.e. $\beta_z^* := \arg_{\beta_1}\left(\partial_{\beta_1}^2 \beta_z^\infty = 0\right)$, which gives

$$\beta_z^* = \beta_0\left(1 + \frac{\epsilon_z}{\epsilon_0}\right) + \frac{1}{2\epsilon_0} \log\left[\frac{1 + \cosh(\beta_{\text{min}}\epsilon_z)}{1 + \cosh(\beta_{\text{max}}\epsilon_z)}\right]. \tag{S9}$$

For small ϵ_z we have that $\beta_z^* \approx \beta_0$, therefore in this regime β_0 specifies the threshold temperature for which the machine changes its regime of operation. Another interesting characteristic of the function β_z^∞ is the slope $\mathcal{A}$ at the threshold point, i.e.

$$\mathcal{A} := \left.\frac{\partial \beta_z^\infty}{\partial \beta_1}\right|_{\beta_1=\beta_z^*} = -\frac{\epsilon_0}{e_z} \cdot \frac{g_{\max} + g_{\min} - 2g_{\max}g_{\min} + 2(1-g_{\min})g_{\min}\sqrt{\frac{g_{\max}(1-g_{\max})}{g_{\min}(1-g_{\min})}}}{g_{\max} - g_{\min}}, \tag{S10}$$

where we used a short-hand notation $g_{\max} := g_z(\beta_{\max})$ and $g_{\min} := g_z(\beta_{\min})$. For example, for a particular choice of parameters $\beta_{\min} = 0$ and $\beta_{\max} = 1$ we have $\mathcal{A} = -(\epsilon_0/e_z)\tanh(\epsilon_z/4) = -\epsilon_0/4 + \mathcal{O}(\epsilon_z^2)$. We therefore see that, for small ϵ_z, the parameter ϵ_0 specifies the slope of the threshold in β_z^∞.

B Details of the thermodynamic neuron model

Consider the $n+1$ qubits $\mathcal{C}_i$ for $i \in \{0, 1, \ldots n\}$ that comprise the collector $\mathcal{C}$ with energies arranged in a vector $\boldsymbol{\epsilon} = (\epsilon_0, \epsilon_1, \ldots \epsilon_n)$ and weakly coupled to heat baths with corresponding temperatures β_i arranged in a vector $\boldsymbol{b} = (\beta_0, \beta_1, \ldots, \beta_n)$. The logical action of the thermodynamic neuron is characterized by a string $\boldsymbol{s}$ with elements ± 1 defined as $\boldsymbol{s} := [(-1)^{h_0 \oplus 1}, (-1)^{h_1 \oplus 1}, \ldots, (-1)^{h_n \oplus 1}]$. The energy of qubit $\mathcal{C}_z$ is chosen to be $\epsilon_z = (\boldsymbol{s} - \bar{\boldsymbol{s}}) \cdot \boldsymbol{\epsilon} = \sum_k (-1)^{h_k} \epsilon_k$. The steady state solution for β_z^∞ satisfies

$$g_z(\beta_z^\infty) = \Delta g_z(\beta_v) + (1-\Delta) g_z(\beta_r), \tag{S11}$$

The virtual temperature β_v satisfies

$$e^{-\beta_v \epsilon_z} = \frac{g_0(h_0) g_1(h_1) \ldots g_n(h_n)}{g_0(h_0 \oplus 1) g_1(h_1 \oplus 1) \ldots g_n(h_n \oplus 1)}, \tag{S12}$$

which implies the that the virtual (inverse) temperature β_v is given by

$$\beta_v = \frac{1}{\epsilon_z} \sum_{i=0}^{n} \log\left[\frac{g_i(h_k \oplus 1)}{g_i(h_k)}\right] = \frac{1}{\epsilon_z} \sum_{k=0}^{n} (-1)^{h_k} \beta_k \epsilon_k. \tag{S13}$$

Proceeding as in Appendix A we can now restrict the range of β_z to $[\beta_{\text{min}}, \beta_{\text{max}}]$ by demanding that Eq. (S5) is satisfied, remembering that now β_v is a linear combination of $n+1$ temperatures. We therefore arrive at the following expression for β_z^∞:

$$\beta_z^\infty = \frac{1}{\epsilon_z} \log \left[Q(\beta_v)^{-1} - 1 \right], \tag{S14}$$

where $Q(\beta_v) := g_z(\beta_{\text{hot}}) g_z(\beta_v) + g_z(\beta_{\text{cold}})(1 - g_z(\beta_v))$ and β_v is the virtual temperature given in Eq. (16). From here one can perform similar types of calculations as in Supplementary Material A.

C Combining thermodynamic neurons using a clock

An alternative approach to combine thermodynamic neurons into networks is to use a timing device (a stopwatch clock). The main observation is that we can ensure correct operation of the device when the layers of thermodynamic neurons are synchronized, i.e. are operating one after the other.

Let us define two relevant time parameters in this context. Firstly, we denote with t_{steady} the time required for the first thermodynamic neuron to reach a temperature that is sufficiently close to its steady state output temperature β_z^∞. Secondly, let t_{const} represent a time interval during which the temperature of the finite thermal environment $\mathcal{B}_z$ can be considered constant when used as the input for the second thermodynamic neuron. These two time parameters can be determined numerically by explicitly solving the machine's dynamics. Crucially, they both depend on the chosen thermalization model.

The gateway operates by successively turning on and off the coupling between the finite heat bath $\mathcal{B}_z$ and the qubit $\mathcal{C}^1$. In the first iteration the coupling is turned off, hence the first thermodynamic neuron reaches its steady state as if it was not connected to any other system. After time t_{steady} the coupling between $\mathcal{B}_z$ and $\mathcal{C}^1$ is turned on for time t_{const} and the second

thermodynamic neuron evolves towards its steady-state. In this time range the heat bath $\mathcal{B}_z$ effectively behaves as an infinite heat bath. After time $t_{\text{steady}} + t_{\text{const}}$ the coupling is turned off and the first thermodynamic neuron is to drive the finite bath $\mathcal{B}_z$ back to the target temperature β_z^{∞}. The cycle is repeated roughly $t_{\text{therm}}/t_{\text{const}}$ times. Consequently, at each time the two thermodynamic neurons operate effectively as two disconnected devices: No unwanted heat currents flow through $\mathcal{B}_z$ and qubit $\mathcal{C}_1'$ is coupled to a heat bath which for all purposes behaves as an infinite heat bath.

The above dynamics requires a timing device which will turn on and off the qubit-bath couplings at appropriate times. Such a dynamics can also be realized autonomously by using an autonomous clock powered by heat baths at different temperatures [42]. Therefore the advantages of clock-based gateway is that it does not require measuring the temperatures of heat baths and the network can (when using an autonomous clock) operate fully autonomously, i.e. without the need of a time-dependent control. The downside of this approach is that it requires a ticking clock (stopwatch) that increases the complexity of model and leads to additional thermodynamic costs. We emphasize that this implementation of a gateway allows for further optimization: By choosing different thermalization models one can vary t_{steady} and t_{const}.
